# Micronized Rose Petal Powder: A Valuable Edible Floral Food Ingredient Containing Bioactive Compounds

**DOI:** 10.3390/molecules29204931

**Published:** 2024-10-18

**Authors:** Renata Różyło, Ryszard Amarowicz, Michał Adam Janiak, Marek Domin, Igor Różyło, Klaudia Rząd, Arkadiusz Matwijczuk, Robert Rusinek, Marek Gancarz

**Affiliations:** 1 Department of Food Engineering and Machines, University of Life Sciences in Lublin, 28 Głęboka Str., 20-612 Lublin, Poland; 2 Department of Chemical and Physical Properties of Food, Institute of Animal Reproduction and Food, Research, Polish Academy of Sciences, Tuwima 10, 10-748 Olsztyn, Poland; r.amarowicz@pan.olsztyn.pl (R.A.); m.janiak@pan.olsztyn.pl (M.A.J.); 3 Department of Biological Bases of Food and Feed Technologies, University of Life Sciences in Lublin, 28 Głęboka Str., 20-612 Lublin, Poland; marek.domin@up.lublin.pl; 4 Faculty of Medicine, Medical University of Lodz, Al. Kościuszki 4, 90-419 Łódź, Poland; rozyloigor@gmail.com; 5 Department of Biophysics, University of Life Sciences in Lublin, 20-950 Lublin, Poland; klaudia.rzad@up.lublin.pl (K.R.); arkadiusz.matwijczuk@up.lublin.pl (A.M.); 6 Institute of Agrophysics, Polish Academy of Sciences, Doświadczalna 4, 20-290 Lublin, Poland; r.rusinek@ipan.lublin.pl (R.R.); m.gancarz@urk.edu.pl (M.G.); 7 Faculty of Production and Power Engineering, University of Agriculture in Krakow, Balicka 116B, 30-149 Krakow, Poland; 8 Center for Innovation and Research on Pro-Healthy and Safe Food, University of Agriculture in Kraków, Balicka 104, 30-149 Kraków, Poland

**Keywords:** micronization, flower petal, rose, phenolic identification, antioxidant activity, infrared spectroscopy-FTIR

## Abstract

Flower petals, as byproducts, provide significant health benefits and can be used in food production. In this study, the impact of the micronization process using a ball mill on the properties of micronized powders derived from wild rose petals of the rugosa variety (*Rosa rugosa Thunb*.) was examined. The micronized rose powders were subjected to an investigation regarding their particle size, color, molecular characterization (FTIR), electronic nose procedure and antioxidant potential. The study found that micronization considerably reduced d50 particle dimensions from 98.6 µm to 39.9 µm. An FTIR analysis revealed the presence of characteristic (2980, 1340, and 1225 cm^−1^) bands. The hydrolysable tannins are the most abundant polyphenolic chemicals in rose powders, followed by anthocyanins. Rose powders are an extremely valuable antioxidant raw material due to their high total phenol content (71.8 mg GAE/g), which increased by approximately 26% after micronization. The antioxidant activity, as determined by ABTS^•+,^ DPPH^•^ and FRAP, is likewise very high. The intensity of volatile chemicals decreased in powders after micronization.

## 1. Introduction

Edible flowers have lately acquired popularity in the production of food due to their functional properties, such as adding color, texture, and expressiveness. They can also be a source of bioactive chemicals. The presence of bioactive chemicals in flowers has been studied and identified in the petals and infusions of calendula, dahlia, rose, and centaurea [1]. Cornflower petals have also been found in studies to be a good source of antioxidants [2,3]. So far, studies on edible flower petals such as rose, sunflower, and calendula flower petals have revealed that these flowers contain essential nutrients for a healthy diet [4]. 

Rose petals have been shown to be high in dietary phytochemicals such as flavonoids (anthocyanins and flavanols), carotenoids, and phenolic acids. The rose is an antioxidant, anti-inflammatory, anti-cancer, anti-aging, and anti-microbial agent due to these components [5]. Research conducted on the petals of rose varieties has demonstrated that they serve as a potent source of antioxidant compounds. The rose petals of the Lovely Red variety were identified to contain fifteen distinct phenolic compounds, such as quinic acid, catechin, **galloyl hexose malic acid**, **myricetin 3,5-di-*O*-glucoside**, **bis-HHDP-hexose**, **galloyl-bis-HHD Phexose**, **quercetin-*O*-pentoside**, **quercetin-*O*-hexoside**, **ellagic acid, rutin**, **quercetin-Orhamnoside**, **quercetin-Ogalloylrhamnoside**, **kaempferol-Opentoside**, **kaempferol-*O*-hexosyldeoxyhexoside**, **kaempferol-Odeoxyhexoside**. Furthermore, the presence of unknown ellagitannins was detected [6].

Rose petals, in addition to anthocyanins and flavonoids, include volatile chemicals [7,8]. Damask rose oils have been shown to contain twenty-five volatile constituents. β-Damascenone, a critical indicator of rose oil quality, was found in twenty-two different genotypes. Nonadecane (42.51%), β-citronellol (40.82%), n-heneicosane (34.69%), geraniol (27.76%), and n-tricosane (14.2%) were the most abundant components in damask rose oil [9]. Volatile concentrates obtained from rose water from Rosa damascena flowers contained volatile substances, which consisted mainly of 2-phenylethanol (69.7–81.6%), linalool (1.5–3.3%), citronellol (1.8–7.2%), nerol (0.2–4.2%) and geraniol (0.9–7.0%) [10]. The compounds found in the highest concentrations in rose petals (Rosa hybrida, cvs David Austin) were phenylethanol, citronellol, nerol, geraniol, and eugenol [11].

Due to the presence of numerous valuable compounds, rose petals ought to be utilized to a greater extent in the production of food and dietary supplements. Rose petals are currently utilized as a major ingredient or additive in the manufacture of jams [12], jellies, biscuits, salads, ice cream, juices, and wine [5]. Products made from rose petals typically include high levels of bioactive chemicals, including anthocyanins, polyphenols, and flavonoids [13], and can thus be used to create novel foods. Because rose petals are a seasonal product, they must be treated to provide a usable raw material [14]. Freeze-drying rose petals is an effective technique for preserving their antioxidant activity and color [15,16].

Following the freeze-drying process, plant materials are typically pulverized into a powder that can be used as a raw material in the creation of functional foods or dietary supplements [17,18]. Previous research has demonstrated that micronization, or the very fine grinding of various plant materials, greatly reduces particle size, which could increase the antioxidant activity of the powders obtained [19]. The effect of the micronization process on particle size and antioxidant activity varies depending on the type of material, and further research is required in this field. Furthermore, there have been no studies on wild rose petals, which is why this topic was examined. By employing FTIR spectroscopy (Fourier Transform Infrared Spectroscopy), it was also possible to examine the impact of the micronization process on changes in the inter-molecular and conformational properties of the obtained powdered end products.

## 2. Results and Discussion

### 2.1. Particle Size Characterization of Micronized Rose Flower Powders

The study showed that using a ball mill to make tiny particles changed the size of the powdered rose samples significantly, as expected (in our previous study [20], where the control sample was raspberry pomace, and due Table 1). During the measurements, the mean particle sizes in volume (D [4;3] [μm]) and mean particle sizes in surface (D [3;2] [μm]) were obtained. The particle sizes were also determined for 10% (D10), 50% (D50), and 90% (D90) of the total sample volume. At a micronization duration of 10 min, the changes in particle diameters were substantial. However, increasing the time had no meaningful impact, but it was statistically significant. The mean particle size (D [4;3]) was equal to 141 μm for the control sample (CR). This dimension was nearly twice as great to the difference in the morphological structure of the examined raw-material, rose petals came apart quite easily after freeze-drying. After 10 min (10MR) of micronization, this value dropped to 90.3 μm, and after 20 min of micronization (20MR), to 81.4 μm. More than 90% of the particles (d90) in the control sample (CR) were smaller than 312 μm. This parameter was 189.3 μm in the sample after 10 min of micronization and 168.7 μm after 20 min of micronization. For CR, the particle size for the 50% share (d50) was less than 98.6 μm, 45.9 μm for 10MR, and 39.9 μm for 20MR. When compared to our prior research on the micronization of raspberry pomace [20], the reduction in this size was enormous, but no such dramatic alterations were found in the case of rose petals. Perhaps they stuck together again after stroking such delicate tissues of rose petals.

### 2.2. Color Parameters of Micronized Rose Flower Powders

**Figure 1:**
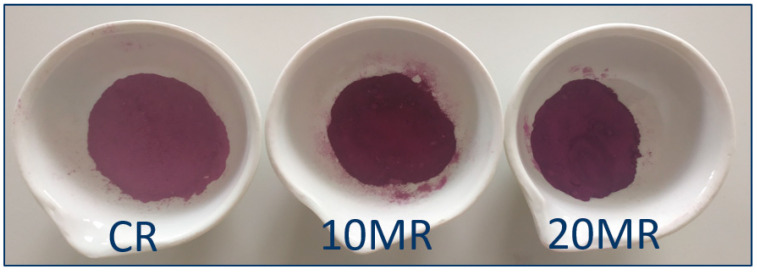
Appearance of control and micronized rose flower powders: CR-control (without micronization) rose powder; 10MR-rose powder micronized for 10 min; 20MR-rose powder micronized for 20 min.

Powders made from rose petals vary greatly in almost all color characteristics (Table 2); only the h parameter, which determines the color angle, was consistent across all powders. The control sample had the highest brightness (L* = 49.9), and as the micronization period increased from 10 min (L* = 33.4) to 20 min (L* = 30.6), the powders darkened (Figure 1). This might be due to the greater packing of smaller particles, resulting in a darker color during the extension of the micronization process. The a* parameter, which determines the share of red color, increased between 21.9 and 28.9 as the micronization period increased from 0 to 20 min. In the event of negative values, the percentage of blue color indicated by the b* parameter grew as the micronization duration rose from 0 to 20 min, in the range of −7.34 to −10.2. Longer-micronized samples had higher color intensity, ranging from 23.1 to 30.7. In the measured range, the BI brownness index increased as the micronization time increased. For parameters L*, a*, b*, and C*, there was a bigger difference between the control sample and the sample micronized for 10 min than between the 10 min and 20 min samples.

When comparing the micronized samples to the control sample, the ΔE parameter was fairly high, averaging 17.1 for the sample micronized for 10 min, and 20.7 for the sample micronized for 20 min. This indicates that the color changes were noticeable to the naked eye. Previous research found that micronized samples of raspberry pomace differed marginally in color from control samples [20], while the color of micronized spinach stems and leaves varied significantly [21]. Wet micronization yielded the most significant results. Other tests found substantial color changes amongst ginger powders with varied particle sizes. According to the authors, color sensations are dependent on the interaction between light and the material surface, which is determined by particle size. The particle size of food powders is an important morphological element that influences the physicochemical qualities of the product [22].

### 2.3. Analysis of the Micronized Rose Petal Powders Using Infrared Spectroscopy-FTIR

In the next stage of the study, FTIR measurements of infrared spectra were performed to evaluate the quality and molecular properties of the micronized rose petals. Table 3 presents the characteristic bands observed in the recorded spectra, which were subsequently analyzed based on literature data and many years of combined experience of our research group, and assigned to corresponding vibrations of specific functional groups [23,24].

In Figure 1, the spectra were also normalized at the wavenumber ~3298 cm^−1^ to emphasize the observed effects and improve the legibility of the presentation.

In the detailed description of the bands present in the obtained FTIR spectra, one should first note the band with the maximum at ~3298 cm^−1^. It is a characteristic band corresponding to the vibrations of hydroxyl groups in the molecules of simple sugars as well as polysaccharides present in the analyzed samples [20,23]. Said groups are involved in the formation of hydrogen bonds between smaller molecules of polysaccharides, constituting the primary building blocks of the samples. Next, we can identify the sharp bands with the maxima at between 3000 and 2800 cm^−1^, which are characteristic of symmetric and asymmetric stretching vibrations in CH_2_ and CH_3_ groups [23]. In the case of samples subjected to micronization, we can observe a very characteristic fading of the band with the maximum at ~2974 cm^−1^, which provides an excellent spectroscopic marker of the treatment process employed in this case. Both in the 10MPR and 20MPR samples, the band faded immediately. As we proceed to the so-called fingerprint region, we can note the first key band with the maximum at ~1732 cm^−1^. It corresponds to vibrations characteristic of carbonyl groups [20,24], both free and hydrogen-bonded, present in the molecules of protein and fatty radicals [20]. The band with the maximum at ~1655 cm^−1^ and primarily 1609 cm^−1^ is the deformation vibration characteristic of the already mentioned hydroxyl groups. Here, we can already clearly note that the band’s intensity increases with the process of micronization. 

**Figure 2:**
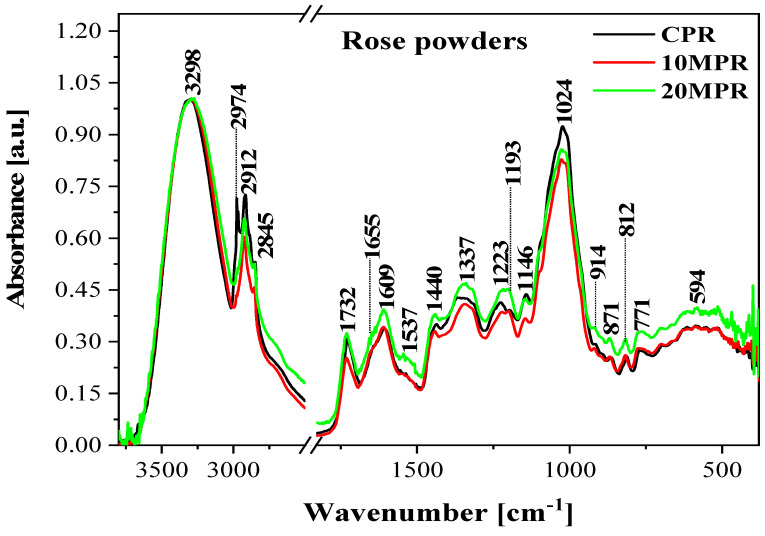
FTIR spectra for the analyzed samples of micronized rose flower powders recorded in the spectral range from 450 to 3600 cm^−1^.

The maximum at 1537 cm^−1^ is characteristic of stretching vibrations of C=C groups and, as we can see in Figure 2, its intensity increases noticeably for the 20 MPR sample [23,28]. The bands with the maxima at ~1440 and 1408 cm^−1^ are characteristic deformation vibrations of CH_2_ groups [29]. Another important region is found at ~1337 cm^−1^. It corresponds to the C-H deformation vibrations enhanced by O-H deformation vibrations. This region is particularly noteworthy, as with the process of micronization, the bands shift toward shorter wavenumbers relative to samples not subjected to the process. The micronization process may lead to the breakup of larger H-bonds, and vibrations of hydroxyl groups may enhance other regions of the bands. The vibrations of those groups occur in polysaccharide molecules (cellulose and hemicellulose) that constitute one of the primary ingredients of the samples. This means that the bands are also an excellent molecular marker of the relevant changes. The same can be said about the next band, with the maximum at approximately 1223 cm^−1^. It is characteristic of a range of vibrations, respectively: deformation-OH (in plane), deformation CH_2_, and deformation C-H [25], as well as stretching in the C-O-C system of cellulose and hemicellulose molecules present in the studied samples. This maximum is also an excellent molecular marker reflecting the process of micronization in a way that is clearly visible in the registered spectra. However, when interpreting the obtained spectra, it should be remembered that FTIR is not a quantitative method, and, in this particular case, peak changes should also be considered by comparing their intensity. The intensity comparison for I_1223_/I_1609_ reveals slight changes, e.g., for the CPR sample, it is ~1.11, while for the 10 MPR sample, it increases to 1.24. This confirms the already described changes in the number of structures between the control samples and samples subjected to micronization. Another region of intensive vibrations is a band with the maximum at approx. 1024, which corresponds to characteristic vibrations of ν(C-O) groups [23], primarily the C-O-C system present in polysaccharide molecules (cellulose, hemicellulose, and lignin) [25]. It is noteworthy that C-C vibrations are present in this region, but they overlap (i.e., interfere) with the mentioned C-O/C-O-C vibrations [26,27]. The intensity of these vibrations also increases with the micronization process employed [20].

Finally, in the region below 950 cm^−1^, it can be noticed that the band’s intensity increases with the time of micronization. It corresponds to one of the characteristic vibrations related to conformational changes in the analyzed material, on β-1,4-glycoside bonds in cellulose molecules and α-1,6-glycoside bonds. This can be associated with changes in the bonds between single mers in larger polysaccharide chains of the analyzed structures [20,24].

To briefly recapitulate the results obtained with the use of FTIR spectroscopy, it could be noted that both the relative changes in the intensity of most bands and clear shifts or fading of some bands are evidence of the significant impact of micronization on the molecular structure of the analyzed samples. It is apparent that the process leads to cleavage of intramolecular hydrogen bonds in the structure of polysaccharides (cellulose, hemicellulose), which results in a significant increase in the presence of amorphous structures [20]. This becomes more apparent depending on the intensity of the micronization process (in terms of its duration). However, the process of micronization also revealed 4 fairly clear marker bands with the maxima at, respectively, approx. 3298, 2974, 1337, and 1223 cm^−1^. In particular, the comparison of band intensities, e.g., I_1223_/I_1609_, demonstrated that FTIR spectroscopy allows one to viably track even slight changes occurring in samples of this type. A comparison of intensity ratios in other regions also yielded similar results; however, a more detailed investigation into this problem is required. As follows from the literature, sample micronization usually leads to the breakup of amorphous regions on an ordered surface of a crystalline substance [20]. As such, the rigid and ordered polysaccharide structure is torn apart and destroyed during grinding. In turn, the noticeable slight change in the bands’ shape also results from the cleavage of hydrogen bonds in the polymeric chain of the sample’s primary building, i.e., its primary stabilizer via hydrogen bonds. It should also be mentioned that the employed process of micronization did not affect the positions of the primary functional groups of the polymer system, which means that the process did not negatively affect the samples’ quality.

### 2.4. Sugar Contents in Micronized Rose Flower Powders

**Figure 3:**
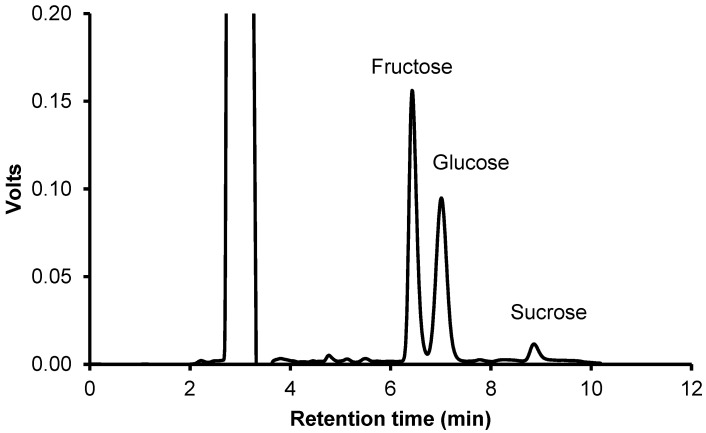
HPLC chromatogram and sugar concentration in micronized rose flower powders.

There was a significant increase in the content of simple sugars in rose flower powders after using the powder micronization process (Table 4). The samples were not significantly different when micronized for 10 and 20 min. The chromatogram and sugar concentration in micronized rose flower powders are shown in Figure 3. The fructose content increased by about 28%, and the glucose content increased by about 17%. No significant changes in the amount of sucrose were observed.

The content of fructose and glucose increased significantly under the influence of micronization, as shown in our previous studies on raspberry pomace powders. These studies revealed that there was a probable break in the intramolecular hydrogen bonds of polysaccharides, which resulted in an increase in the share of simple saccharides in the mixture [20]. Other authors suggest that changes in the content of simple sugars could be caused by the destruction of cell membranes during intensive grinding of particles in a ball mill [30]. It is possible that the reduction in particle size resulted in an increase in the extraction of these compounds. Research conducted on soy fibers has demonstrated that, as a result of micronization, the number of polysaccharides is reduced [31].

### 2.5. Identification of Phenolic Compounds in Micronized Rose Flower Powders

The list of the identified compounds is given in Table 5. Rose petals contain a range of ellagic acid derivatives as well as anthocyanidin compounds.

**Figure 4:**
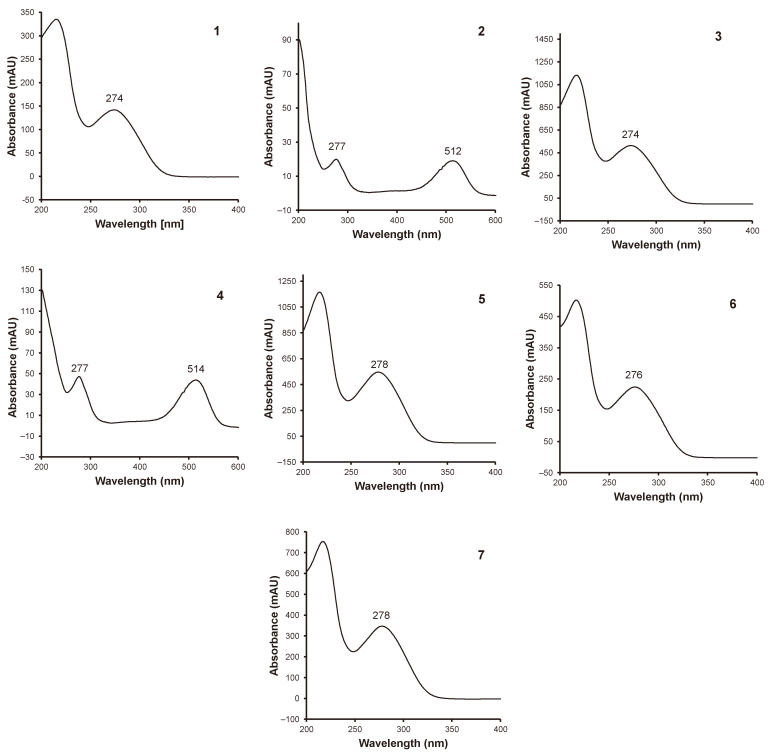
UV and UV-VIS absorbance spectra of individual phenolic compounds and identification of the main phenolic compounds of rose petal powders by UHPLC-QTOFMS/MS; **1**—**Bis-HHDP-hexose isomer**; **2**—**Cyanidin 3,5-di-*O*-glucoside**; **3**—**Bis-HHDP-hexose isomer**; **4**—**Peonidin 3,5-di-*O*-glucoside**; **5**—**Galloyl-bis-HHDP-hexose isomer**; **6**—**Galloyl-bis-HHDP-hexose isomer**; **7**—**Galloyl-bis-HHDP-hexose isomer**. **HHDP-hexahydroxydiphenoyl**.

Peaks 1, 3, 5, 6 and 7 can be attributed to bis-HHDP (**hexahydroxydiphenoyl)-hexose**, **galloyl-bis-HHDP-hexose isomers**; peaks 2 and 4 to **cyanidin 3,5-di-*O*-glucoside** and **peonidin 3,5-di-*O*-glusocide** (Figure 4). The presence of gallic acid derivatives, as well as anthocyanins in flowers, was also reported in Rosa rugosa petals [32] and in Rosa damascena [33]. Cendrowski et al. [32] observed the presence of sanguine H-2 (m/z 1103), unknown ellagitannins with molecular ions 860, 937, 1105 and isomer of **galloyl-bis-HHDP glucose** (m/z 935), as well derivatives of cyanidin and peonidin. Among those compounds, **cyanidin 3,5-di-*O*-glucoside** and **peonidin 3,5-di-*O*-glusocide** were the dominant ones which is in accordance with our study. Mohsen et al. [33] showed that a range of ellagic acid derivatives is present in rose petals. Compounds characterized by molecular ions 633, 765, 783, 785, 787, 934, 935, etc. are present and were attributed to ellagic acid derivatives such as **galloyl-HHDP-hexose**, **digalloyl-hexosyl-ellagic acid**, **bis-HHDP-hexose**, **di-*O*-galloyl-HHDP-hexose**, **tetra-*O*-galloyl-hexose**, unknown ellagitannin as well as **galloyl-bis-HHDP-hexose**. Dias et al. [34] reported the presence of compounds with recorded molecular ions of 783 and 935. For those two compounds λmax were recorded as 276 and 278, respectively, which is similar to our findings. Dias et al. [33] also reported the presence of m/z 1567 (sanguiin h10) with λmax 278. Fragmentation of this compound revealed m/z 935. It might suggest that in the case of the samples assessed in this study, some of the compounds may also be, in fact, products of cleavage of the bigger structure, which was reported by Cendrowski et al. [32] (m/z 1103 > 935). Results presented in our study demonstrated that hydrolysable tannins are dominant polyphenolic compounds in rose petals, followed by anthocyanins.

### 2.6. Total Phenolics and Antioxidant Potential of Micronized Rose Flower Powders

Table 6 summarizes the total phenolic and antioxidant potential of micronized rose flower powders. The content of total phenolics in micronized rose flower powders increased significantly by approximately 26% after the micronization process. There were no significant differences between 10 and 20 min of micronization. In previous studies in which we examined raspberry pomace powders [20] there was also a significant but not as large of an increase (15%) in total phenolics. It should be noted that rose petal powders are a very valuable antioxidant raw material due to the very high content of total phenolics, i.e., 71.8 mg GAE/g (control sample); this value is 3.6 times higher than that found for raspberry pomace powders in previous studies. Micronized rose petals powders had a total phenolic content ranging from 90.5 to 93.3 mg GAE/g. The obtained values were over two times higher than in the case of rose petals tested by other authors [35]. In our studies, rose petals were freeze-dried, while infrared drying was used in others. According to the researchers [35], different drying processes and conditions can have a considerable impact on the amount of active chemicals in plant materials. In addition, other varieties of roses were tested. Other authors [6] noticed that freeze and hot air-drying methods had similar effects on the retention of total phenolics and antioxidant activity, as well as the color of different cultivars of rose petals, with freeze drying retaining more red color.

The test findings indicated that the rose petal powders had outstanding antioxidant activity. Other scientific research has demonstrated that rose flower petals are an important compound with excellent antioxidant capabilities and significant nutraceutical potential [36]. After micronization, the antioxidant potential assessed in our study by ABTS, DPPH, and FRAP increased significantly. There were no significant variations in ABTS micronization times between 10 min. and 20 min. Ten minutes of micronization increased the ABTS value by 30%. The remaining DPPH and FRAP indicators showed enhanced activity with increasing micronization time. However, the differences between the samples micronized for 10 and 20 min were not as significant as those between the control sample and the sample micronized for 10 min. When compared to the control sample, the DPPH index increased by 37% after 10 min of micronization and 42% after 20 min.

The FRAP index’s value increased by 22 and 34% after 10 and 20 min of micronization, respectively. Previous research [20] on raspberry pomace powders showed a considerable rise in ABTS (22%) and FRAP (20%) after only 10 min of micronization. Prolonging micronization had no meaningful effect on these indicators. The DDPH index evaluated for raspberry powders showed a small drop in value. Other methods of raspberry fruit micronization [37] involving fluidized bed jet milling similarly increased the antioxidant activity of the studied powders. In the case of grape pomace powders [38], a drop in antioxidant capacity was seen following superfine grinding, which the authors attribute to a large increase in temperature during this procedure. In our investigations, the micronization period was rather short, and there was no major temperature increase. Other investigations found that ultra-fine grinding under cryogenic conditions considerably improved wheat bran’s antioxidant activity [39]. Gong et al. [40] found that superfine grinding resulted in a considerable increase in the antioxidant activity of mushroom powders.

### 2.7. Electronic Nose Research Results

Six of the eight sensors installed responded to the presence of volatile organic compounds in the tested samples (Table 7). The highest value was obtained for the 2602 sensor for CR, and the lowest value for AMS-MLV-P2 for 20MR. Generally, CR samples have the highest intensity, and 20MR samples have the lowest intensity. The results show that micronization affects the emission of volatile organic compounds from the material that has been subjected to this process. The longer the micritization process, the greater the decrease in the intensity of volatile organic compound emission.

Evidence that the parameters of various types of processes to which materials are subjected influence changes in the intensity of volatile organic compound emissions. In this case, the responses of the electronic nose respond to the intensity of volatile substances of various samples, which is also confirmed by tests of other materials, for example, coffee [41], corn groats [42] and corn [43].

### 2.8. Principal Component Analysis (PCA)

**Figure 5:**
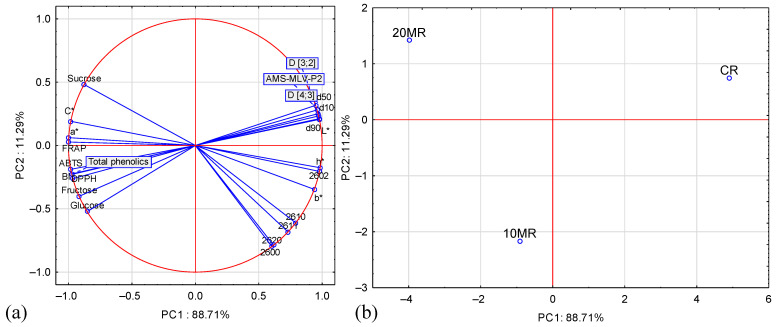
Projection of variables parameters on the PC1 and PC2 loadings plot—(**a**), and projection of sample type on the PC1 and PC2 scores plot—(**b**).

The projection of the cases onto the PC1 and PC2 plane (Figure 5a,b) indicates that both main components differentiate the examined cases. The first principal component, PC1, explains 88.71% of the variability of the system and differentiates the absence of MR (positive PC1 values) and the presence of MR (negative PC1 values). In turn, the second principal component of PC2 describes the differences between 10MR (negative PC2 values) and 20MR (positive PC2 values) in 11.29%.

The PCA also showed a strong and positive correlation between: D [3;2], D [4;3], d10, d50, d90, L*, AMS-MLV-P2, and CR (Figure 5a,b). A strong and positive correlation with sucrose, C* and 20MR was also demonstrated. In turn, between, D [3;2], D [4;3], d10, d50, d90, L*, AMS-MLV-P2, and sucrose, C*, a*, FRAP, ABTS, BI, total phenolic, DPPH, fructose, glucose showed a strong but negative correlation. Also, a strong and negative correlation was demonstrated between the responses of sensors 2600, 2602, 2610,2611,2620, h*, b* and the sucrose, C*, a*, FRAP parameters for 20MR. This means that micronization causes, among other things, a darker color of the micronized material and increases its sugar content.

## 3. Materials and Methods

### 3.1. Materials

Rose petals from the rugosa (*Rosa rugosa Thunb*.) came from the wild areas. After harvest, the rose petals were frozen at −30 °C and then freeze-dried (pressure 20 Pa time 72 h, Christ Alpha 2–4 LD plus device).

### 3.2. Micronization of Freeze-Dried Rose Flower Powders

The freeze-dried rose flower petals were first crushed for 5 s in a knife grinder, then micronized for 10 and 20 min in a ball mill at 600 rpm, using a Pulverisette 6 Fritsh (Idar-Oberstein, Germany) [20]. The bowl of the ball mill was filled with 15 steel balls. The micronization process was repeated three times with the same sample mass, 50 g. After micronization, the powders were brought to room temperature, weighed, vacuum packaged in 5 g, and stored until all measurements were completed. During the initial tests, the micronization time was determined, resulting in a significant variance in the particle sizes of the samples. Following micronization, the particle size and temperature were monitored simultaneously. It was found that 10 min of micronization greatly reduced particle size. Extending the micronization time to 10 min raised the raw material’s temperature to 38 °C, then 53 °C after 20 min. We did not want to induce significant degradation of the chemicals. Therefore, we completed the process after 20 min. Consequently, we obtained three distinct assortments of powdered rose petals: freeze-dried and pre-crushed on a knife grinder referred to as control rose petals (CR), micronized for a duration of 10 min (10MR), and micronized for a duration of 20 min (20MR).

### 3.3. Particle Size Analysis

The analysis of the particle size of the powdered rose petals was conducted on a laser analyzer Mastersizer 3000 (Worcestershire, UK) equipped with a dry dispersion attachment (Aero S), employing the previously outlined methodology [21].

### 3.4. Color Evaluations

The samples that were analyzed were measured using a 4Wave CR30–16 colorimeter (Tychy, Poland), using the CIE L* a* b* scale. On this scale, the brightness of the material was represented by the parameter L*, which ranged from 0 to 100. The color index a*, which varies from −150 to +100, represents the proportion of green with negative values and red with positive values. The color index b*, which spans from −150 to +150, represents the proportion of blue with negative values and yellow with positive values. C* (chroma) denotes color intensity, and h (angle) denotes color angle. Moreover, the determination of the color change (ΔE) and the browning index (BI) was performed in accordance with the principles of Subhashree et al. [44].

### 3.5. ATR-FTIR Spectra Measurements

An IRSprit spectrometer by Shimatzu (Tokyo, Japan) was used to measure the ATR-FTIR for the analyzed samples. A Zn Se crystal with adequate geometry (45°) was used as an ATR (Attenuated Total Reflection) attachment to multiply the internal reflections of the laser beam. Micronized samples of powdered rose petals were placed on the crystal. The spectrometer attachment considerably improved measurement precision by permitting exact control of the contact between the crystal and the sample, as well as facilitating pressure adjustment. The measurement entailed a total of 24 scans for each sample. The software was then used to automatically average the obtained spectra. Before and after each measurement, the crystal was thoroughly cleaned with ultrapure solvents. The solvents were purchased from Sigma-Aldrich, a company from Poznań, Poland. The scans were taken in the spectral range from 450 to 3600 cm^−1^, at the resolution of 2 cm^−1^. Additionally, each of the spectra was averaged with five prior measurements to avoid problems related to sample homogeneity. The measurements were conducted at room temperature. For better legibility, the spectra were also normalized at the maximum of the vibration corresponding to the hydroxy group. All measurements were conducted at the Molecular Biophysics Institute of the University of Life Sciences in Lublin. The spectra were processed and prepared for publication using Grams AI software (version 9.1) from ThermoGalactic Industries (San Jose, CA, USA).

### 3.6. Determination of Sugars

Sugars were extracted from raspberry seeds with hot 85% (*v/v*) methanol [30,45]. Individual sugars were determined using the HPLC method. Individual sugars were separated using an HPLC Shimadzu system (Shimadzu, Kyoto, Japan), which consisted of an SCL-10A controller, an LC-10AD pump, and a RID-10A detector. A portion of 20 µL of the extract was injected into a Luna Omega 3 µm SUGAR column (4.6 × 250 mm) (Phenomenex, Torrance, CA, USA). The flow rate of the mobile phase (acetonitrile–water, 25:75, *v/v*) was 1 mL/min. For calibration, the external standard method was used.

### 3.7. Determination of Total Phenolic Compounds

The determination of the total phenolic compounds present in the extract was conducted by utilizing Folin–Ciocalteou’s phenol reagent [46]. The final result was portrayed in the form of gallic acid equivalents per gram of rose petal powder.

### 3.8. Analyzing Phenolic Compounds Using HPLC-DAD

The analysis of polyphenolic compounds was conducted by employing RP-HPLC-DAD. Extracts were injected (1 µL) into the Shimadzu Nexera system (Shimadzu, Kyoto, Japan), which consisted of a degassing unit (DGV-20A 5R), two pumps (LC-30AD), an autosampler (SIL-30AC), a column oven, and a PDA detector (SPD-M30A) and a controlling unit (CBM-20A). The flow rate was set to 1 mL per mL. The separation process was conducted using a Kinetex machine from SHIM-POL, Warsaw, Poland, with a C18 2.6 m, 100 A, and a 75 × 3 mm diameter. The separation was monitored at wavelengths of 280 and 520 nm and was conducted under a binary gradient condition. Two types of eluents were used in the analysis, i.e., A and B. Eluent A included water: acetonitrile: trifluoroacetic acid in the proportions 95:5:0.1 (*v*/*v*/*v*), and eluent B included acetonitrile: trifluoroacetic acid in the proportions 100:0.1 (*v*/*v*). The gradient for eluent B was set as follows: 0–10 min: 0–18.8%; 10.5 min: 0%; 12 min: 0%. The peak areas of ellagic acid and cyaniding-3-glucoside were compared to those of prepared calibration curves for the two compounds. The results were given in milligrams of the standard for every gram of extract for every gram of D.W.

### 3.9. Identification of Phenolic Compounds

An Exigent microLC 200 system coupled with a TripleTOF 5600+ mass spectrometer was used to identify more of the compounds from rose petal powders. The electrospray ionization process was carried out in both positive and negative directions. The operating MS conditions were as follows: ion spray voltage of 4.5 kV, turbo spray temperature of 350 °C, flow rate of nebulizer gas (GS1) and curtain gas (GS2) of 30 L/min, declustering potential (DP) and collision energy (CE) for the full-scan MS of 90 or 90 V and 10 or 10 eV, respectively, and for MS2 (MS/MS) mode of 80 or 80 V and 30 or 30 eV. The TOF MS scan was performed at a mass range of 100–1250 m/z. An Exigent Halo C18 column (0.5 × 50 mm, 2.7 m; AB Sciex) was used to separate the compounds. The binary gradient that was employed comprised 0.1% (*v*/*v*) formic acid in water (eluent A) and 0.1% (*v*/*v*) formic acid in acetonitrile (eluent B). It was established from 5 to 90% B within 3 min, maintained to 3.8 min, and maintained to 5% within 4 min, ultimately achieving a duration of 5 min.

### 3.10. Antiradical Activity

The antiradical activity against ABTS^•+^ and DPPH^•^ was determined using the methods described by Re et al. [47] and Amarowicz et al. [48,49]. The results were expressed as millimoles of Trolox equivalents (TE) per gram of powder. The method of Benzie and Strain [50] was used for the determination of ferric-reducing antioxidant power (FRAP). The results were expressed as mmol Fe^2+^ per gram of rose petal powder.

### 3.11. Photochemiluminescence Assay

A photochemiluminescence (PCL-ACL) method was used to evaluate the scavenging activity of rose petal powder samples, in which superoxide radical anions (O_2_^•−^) are generated from luminescence. The reactions were conducted by utilizing kits from Analytic Jena, located in Jena, Germany. The experiment was carried out on a Photochemical device with the help of PCLsoft 5.1 software (Analytic Jena).

### 3.12. Electronic Nose Procedure

Samples of each material with the same mass, 0.5 g, were used to test the emission of volatile substances. Each sample was placed in an Eppendorf tube. Three tubes for each type of material. The emission surface was the same for each sample. This made it possible to maintain constant test conditions for each sample. For each type of material, tests were performed in triplicate. The Agrinose device designed and constructed at the Institute of Agrophysics of the Polish Academy of Sciences in Lublin was used in the study. It has a matrix of eight MOS sensors. Table 8 presents the types and technical data of Agrinose sensors. Seven of them (TGS type) were produced by Figaro Engineering (Japan) and one by Ams (USA). A measurement cycle according to the sampling protocol consisted of a 10 s baseline purge, a 100 s sample draw-in, and a 100 s laboratory air purge. Analog signals were converted to digital signals by means of the software of Agrinose. Obtained sensorgrams were converted to the ∗.xls format and analyzed using the software Statistica (version 12.0, StatSoft Inc., Tulsa, OK, USA).

### 3.13. Statistical Analysis

In order to determine the accuracy of the measurements, means and deviations were calculated, and other statistical analyses were performed in Statistica 12.0 (StatSoft, Kraków, Poland). We used ANOVA and Tukey’s test to see if there were any differences (*p* < 0.05) between the means. The significance of differences (*p* < 0.05) between the means was noted with different letters. Principal component analysis was used to determine the relationships between the studied cases and parameters. Principal components analysis (PCA), analysis of variance and correlation determination were performed at the significance level of α = 0.05. The matrix of data used for the PCA statistical analysis of research results had 24 columns and 3 rows. The Cattel criterion was used to determine the number of principal components in the analysis in both cases, and the input matrix was automatically scaled. All measurements were performed in triplicate.

## 4. Conclusions

In summary, the findings of the experiments proved the feasibility of utilizing a ball mill for the micronization process, which involves the very fine grinding of freeze-dried wild rose petals. After 10 min of micronization, there was a considerable reduction in particle size from d50 = 98.6 μm to d50 = 45.9 μm; thereafter, these changes were less pronounced. Despite the fact that the color of the powders significantly darkened following the treatment, micronized wild rose petal particles exhibited good coloration (with a high share of red and blue components).

In turn, the spectroscopic FTIR analysis revealed the most visible spectral changes in the bands with the maxima at, respectively, ~3298, 2974, 1337,1223 cm^−1^, as well as a general increase in band intensity in the region below 1700 cm^−1^ corresponding to the increased intensity of the micronization process. Band shifts were also evidenced in the aforementioned ranges. The process of micronizing rose petal powders, particularly at higher mill speeds, most likely causes cleavage of intramolecular hydrogen bonds responsible for the stabilization of the primary structure of polysaccharide molecules. Consequently, micronization significantly increases the number of simple sugar molecules present in the system. This change is fairly clearly reflected by the change in the intensity of bands below 950 cm^−1^. It has been determined that rose petals and micronized powders contain a variety of ellagic acid derivatives as well as anthocyanidin chemicals. Among those chemicals, **cyanidin 3,5-di-*O*-glucoside** and **peonidin 3,5-di-*O*-glusocide** were the most prominent. Freeze-dried rose petals are a valuable raw material due to their high total phenolics concentration (71.8 mg GAE/g), and antioxidant activity measured by ABTS^•+^ (0.876 mmol TE/g), DPPH^•^ (0.820 mmol TE/g) and FRAP (1.595 mmol Fe^2+^/g). The micronization process already at 10 min significantly increased total phenolics and antioxidant activity parameters (ABTS^•+^; DPPH^•^; FRAP) by 26%, 30%, 37% and 22%, respectively. Although the intensity of volatile substances decreases after micronization, odorlessness is often desired in food additives. Because of the properties listed above, particularly the antioxidant activity, micronized powders from wild rose petals are recommended as valuable functional additives to a variety of foods.

## Figures and Tables

**Table 1:** Particle size characteristics of micronized rose flower powders.

Name of Sample	D [3;2] (µm)	D [4;3] (µm)	d10 (µm)	d50 (µm)	d90 (µm)
CR	65.1 ± 1.27 ^a^	141.0 ± 6.48 ^a^	31.7 ± 0.65 ^a^	98.6 ± 3.10 ^a^	312.0 ± 19.61^a^
10MR	19.1 ± 0.49 ^b^	90.3 ± 0.71 ^b^	12.8 ± 0.14 ^b^	45.9 ± 0.65 ^b^	189.3 ± 7.48 ^b^
20MR	17.8 ± 1.00 ^b^	81.4 ± 4.37 ^c^	10.2 ± 0.21 ^c^	39.9 ± 0.32 ^c^	168.7 ± 10.61^c^

Values in the same column marked with different letter differ significantly (*p* < 0.05).

**Table 2:** Color parameters of micronized rose flower powders.

Kind of Sample	L*	a*	b*	C*	h*	ΔE	BI
CR	49.9 ± 0.33 ^a^	21.9 ± 0.12 ^a^	−7.3 ± 0.02 ^a^	23.1 ± 0.08 ^a^	341.3 ± 0.04 ^a^	–	15
10MR	33.4 ± 0.18 ^b^	26.1 ± 0.04 ^b^	−8.3 ± 0.13 ^b^	26.8 ± 0.05 ^b^	340.9 ± 0.72 ^a^	17.1	25
20MR	30.6 ± 0.42 ^c^	28.9 ± 0.18 ^c^	−10.2 ± 0.34 ^c^	30.7 ± 0.26 ^c^	340.5 ± 0.50 ^a^	20.7	26

a, b, c—Values in the same column marked with different letter differ significantly (*p* < 0.05).

**Table 3:** Maxima of the FTIR absorption bands and assignment of respective vibrations in the samples of micronized rose petals, corresponding to data in Figure 1; registered in the spectral range of 450–3600 cm^−1^ [20,23,25,26,27].

FTIR	Type and Origin of Vibrations
Band Position [cm^−1^]	
3298	*(intra-)*molecular hydrogen bonding and ν(O-H) in H_2_O and polysaccharide molecules
2974 2906	*asymmetrical* and *symmetrical*: ν(C-H) in CH_2_ and CH_3_ groups
2873 2849	
1732	ν(C=O) *free and hydrogen-bonded*
1655	ν(C=C) and δ(O-H) adsorbed H_2_O
1609	
1537	ν (C=C)
1440 1408	δ (CH_2_) and δ (C-H) significantly enhanced by δ (-OH *in plane*)
1367 1337	Δ(O-H), mainly from deformation C-H
1225 1193	δ(C-H) and asymmetrical bridge oxygen stretching -OH *in-plane* bending
1146	ν(*C-O-C*) and very strong stretching vibrations of *C-O* and vibrations in polysaccharide systems and stretching vibrations of *C-C*
1024 with band enhancements on both sides	
914 871 812	*β*-linkage of cellulose/ring breathing and asymmetrical *out of phase* stretching -OH *out-of-plane* bending and CH_2_ rocking
762 594	

s—symmetric, as—asymmetric, st—strong, w—weak, ν—stretching vibrations, δ—deformation vibrations.

**Table 4:** Sugar contents in micronized rose flower powders.

Sample	Fructose (mg/g)	Glucose (mg/g)	Sucrose (mg/g)
CR 10MR 20MR	132.9 ± 0.5 ^b^ 171.0 ± 3.2 ^a^ 168.2 ± 1.2 ^a^	77.9 ± 0.2 ^b^ 91.6 ± 1.7 ^a^ 88.7 ± 1.9 ^a^	5.97 ± 0.54 ^a^ 6.13 ± 0.24 ^a^ 6.74 ± 0.69 ^a^

a, b, c—Values in the same row marked with different letter differ significantly (*p* < 0.05).

**Table 5:** Identification of the main phenolic compounds of rose petal powders by UHPLC-QTOFMS/MS.

Number	Compound	Ionization	MS	MS/MS
1 2 3 4 5 6 7	**Bis-HHDP-hexose isomer** **Cyanidin 3,5-di-*O*-glucoside** **Bis-HHDP-hexose isomer** **Peonidin 3,5-di-*O*-glucoside** **Galloyl-bis-HHDP-hexose isomer** **Galloyl-bis-HHDP-hexose isomer** **Galloyl-bis-HHDP-hexose isomer**	[M-H]^−^ [M-H]^+^ [M-H]^−^ [M-H]^+^ [M-H]^−^ [M-H]^−^ [M-H]^−^	783 611 783 625 935 935 935	301 449, 287 301 463, 301 633, 301 633, 301 633, 301

HHDP-hexahydroxydiphenoyl.

**Table 6:** Total phenolics and antioxidant potential of micronized rose flower powders.

Antioxidant Potential	CR	10MR	20MR
Total phenolics (mg GAE/g) ABTS (mmol TE/g) DPPH (mmol TE/g) FRAP (mmol Fe^2+^/g)	71.8 ± 1.4 ^b^ 0.876 ± 0.004 ^b^ 0.820 ± 0.019 ^c^ 1.595 ± 0.013 ^c^	90.5 ± 1.7 ^a^ 1.139 ± 0.015 ^a^ 1.124 ± 0.023 ^b^ 1.938 ± 0.031 ^b^	93.3 ± 1.2 ^a^ 1.192 ± 0.019 ^a^ 1.168 ± 0.008 ^a^ 2.139 ± 0.024 ^a^

a, b, c—Values in the same row marked with different letter differ significantly (*p* < 0.05).

**Table 7:** Results (with standard deviation ±) of measuring the intensity of the impact of volatile substances by Agrinose (ΔR/Rmax).

Sample	2602	AMS-MLV-P2	2610	2611	2620	2600
CR	3.40 ± 0.21^a^	0.47 ± 0.12 ^a^	1.45 ± 0.09 ^a^	1.56 ± 0.28 ^a^	1.91 ± 0.29 ^a^	1.80 ± 0.26 ^a^
10MR	2.41 ± 0.58 ^b^	0.29 ± 0.04 ^b^	1.42 ± 0.24 ^a^	1.51 ± 0.14 ^a^	1.69 ± 0.22 ^b^	1.59 ± 0.20 ^b^
20MR	1.30 ± 0.41 ^c^	0.27 ± 0.06 ^c^	0.73 ± 0.22 ^b^	0.71 ± 0.21 ^b^	0.95 ± 0.25 ^c^	0.84 ± 0.21 ^c^

a, b, c—Values in the same column marked with different letter differ significantly (*p* < 0.05).

**Table 8:** Technical data of Agrinose sensors.

Type	Description	Detecting Range (ppm)
TGS2600–B00	General air contaminants, hydrogen, and carbon monoxide	1–3 (H_2_)
TGS2610–C00	LP gas, butane	500–10,000
TGS2602–B00	Ammonia, Hydrogen sulfide (high sensitivity to VOC and odorous gasses)	1–30 (EtOH)
TGS2611–C00	Natural gas, methane	500–10,000
TGS2603–B01	Odors generated from spoiled foods	1–10 (EtOH)
TGS2612–D00	Methane, propane, and butane	1–25%LEL
TGS2620–C00	Solvent vapors, volatile vapors, alcohol	50–5000
AS–MLV–P2	CO, butane, methane, ethanol, hydrogen. Specifically designed for volatile organic compounds (VOCs)	10–10,000

## Data Availability

The data presented in this study are available on request from the corresponding authors.
